# Migration Measurement of Pins in Postoperative Recovery of the Proximal Femur Fractures Based on 3D Point Cloud Matching

**DOI:** 10.3390/medicina57050406

**Published:** 2021-04-22

**Authors:** Kaifeng Liu, Kouki Nagamune, Keisuke Oe, Ryosuke Kuroda, Takahiro Niikura

**Affiliations:** 1 Department of Human and Artificial Intelligent Systems, Graduate School of Engineering, University of Fukui, Fukui 910-8507, Japan; 2 Department of Orthopaedic Surgery, Kobe University Graduate School of Medicine, Kobe 650-0017, Japan; nagamune@u-fukui.ac.jp (K.N.); keisukeo@med.kobe-u.ac.jp (K.O.); kurodar@med.kobe-u.ac.jp (R.K.); tniikura@med.kobe-u.ac.jp (T.N.)

**Keywords:** 3D matching, CT images, internal fixation, migration of implants, proximal femur fractures

## Abstract

*Background and objectives:* Internal fixation is one of the most effective methods for the treatment of proximal femur fractures. The migration of implants after the operation can seriously affect the reduction of treatment and even cause complications. Traditional diagnosis methods can not directly measure the extent of displacement. *Methods:* Based on the analysis of Hansson pins, this paper proposes a measurement method based on three-dimensional matching, which uses computerized tomography (CT) images of different periods of patients after the operation to analyze the implants’ migration in three-dimensional space with the characteristics of fast speed and intuitive results. *Results and conclusions:* The measurement results show that the method proposed in this paper has more minor errors, more flexible coordinate system conversion, and more explicit displacement analysis than the traditional method of manually finding references in CT images and measuring displacement.

## 1. Introduction

Proximal femur fractures commonly occur worldwide, especially in the elderly [1]. With increasing incidence, the number of patients is expected to double by 2025 compared to 1990 [2]. Among these patients, the female population is two times more than the male population [3]. In nearly half of those hip fractures, intracapsular fractures of the femoral neck were observed [4].

Currently, for treating Garden I and Garden II stable femoral neck fractures, the use of internal fixation has become a consensus among orthopedic surgeons [5,6,7]. With internal fixation, the risk of infection, dislocation, femoral fracture, and laxity is lower than with total hip arthroplasty (THA) [8]. Additionally, internal fixation surgery provides minimal invasiveness and significantly reduces postoperative hospitalization time [9]. Various options are currently available for stabilizing the internal fixation of femoral neck fractures, including cannulated screws, dynamic hip screws, proximal femoral locking plates, and other implants. The published literature suggests that implant failure is one of the main reasons for reoperation after internal femoral fixation [10]. Consequently, selecting the appropriate internal fixation solution for the patient to ensure postoperative implant stability with a minimum of movement is critical to the success of the procedure.

The current approach to studying the stability of internal fixation solutions is mainly biomechanical simulation. Researchers have used stress testers to simulate human gait behavior by applying pressure to synthetic bones or cadaveric femora with internal fixation and calculating the implant displacement as a stability criterion [11,12,13,14]. The use of finite element models is also one of the standard methods to simulate implants’ mechanical properties such as stress, strain, the load to failure, and displacement [15,16,17]. The majority of the published literature evaluating internal fixation solutions utilizes simulation models created with a limited number of parameters. These models, therefore, only simulate the forces and displacements of the femur and implant in a particular situation. Nevertheless, during the patient’s postoperative recovery, the femur is subjected to a complex combination of forces that vary with the movement’s posture. Conventional biomechanical simulation results, accordingly, do not provide a comprehensive evaluation of the implant. 

Besides, femoral neck fractures require a lengthy recovery period after surgery, especially in the elderly, who have slow bone healing. Computerized tomography (CT) medical images are simple to obtain and can be acquired at all postoperative time points. It can provide the most direct information about the implant’s displacement under realistic postoperative motion conditions of the patient, which is not easy to achieve with traditional mechanical simulation and finite element methods. To obtain the displacement data of the target object inside the patient, the plastic surgeon primarily selects rigid references in the radiographic images and measures the target object’s position relative to the reference object in different image sequences, respectively. The position coordinates are used to calculate the displacement of the target object in the different image sequences. Such a method is complicated to operate and vulnerable to the subjective factors of the surgeon. In this paper, we take Hansson pins as the object of study and propose a method for the fine registration of rigid references at the fracture site using point cloud information from the 3D reconstruction. The method uses the converted point cloud coordinates to calculate the implant’s displacement and rotation during the postoperative recovery process, and the output uses a 3D model to visualize the displacement results. The importance and originality of this study are that this research addresses the limitations of traditional methods, such as biomechanical simulations’ limitation in simulating patient behavior, and the inability to quantify the displacement rapidly and accurately during diagnosis using CT medical images. The experimental results show that, compared with the traditional method of manual displacement measurement, this method can significantly simplify manual displacement measurement and reduce the measurement time without decreasing the measurement accuracy.

## 2. Materials and Methods

### 2.1. Patients

**Figure 1:**
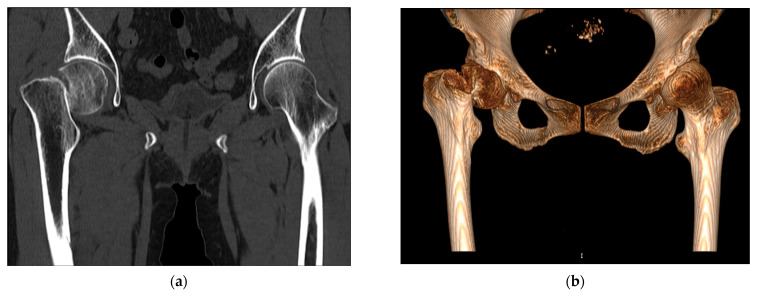
Intracapsular fracture occurring because of low-energy falls in the elderly: (**a**) CT diagnostic image; (**b**) 3D reconstruction model.

We analyzed 10 cases from March 2012 to January 2015 provided by Hyogo Prefectural Awaji Medical Center. All the cases were associated with intracapsular fracture and the age of the patients ranged from 65 to 85. As shown in Figure 1, an intracapsular fracture refers to the femoral neck fracture that occurs within the capsule of the hip joint.

**Figure 2:**
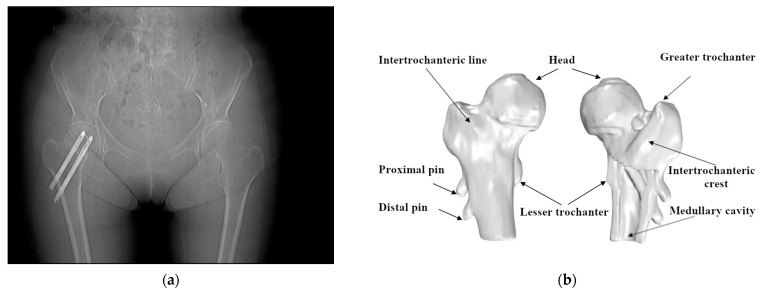
Use of the Hansson pin for the fixation of femoral neck fractures: (**a**) the anteroposterior/posteroanterior X-ray (AP X-ray) of intracapsular fracture fixation using two Hansson pins; (**b**) the position of the Hansson pins in the anatomy of the femur.

Due to the lack of periosteum and limited callus formation, the processing of healing is slow. Among the cases in this research, the Hansson pin (Hansson Pin System, Stryker) [18] was used to fix the femur’s neck. The Hansson pin is a 6.5-mm diameter unthreaded nail with various specifications and has a length ranging from 70 mm to 120 mm. Each pin can be divided into two parts, the outer sleeve and the inner movable hook pin. Typically surgeons use two pins for fixing and screwing out the hook pins when fixing (Figure 2).

We used AP X-rays and computed tomography (CT) to record the patient’s intracapsular fracture status and the Hansson pin’s position at three different times. The time points of recording were preoperative, postoperative, and after one year of recovery, respectively. 3D reconstruction was then performed to assess the patient’s recovery status as well as to calculate the change in Hansson’s pin position.

### 2.2. Image-Based Measurement Method

The traditional method of measuring the migration of a Hansson pin is similar to measuring a broken bone’s displacement after a fracture. A CT scan is performed on the target position. The two-dimensional image measurement software is used to find the landmarks in the CT images, then the landmarks are used to establish a coordinates system. Generally, the surgeon selects the area containing the greater trochanter, lesser trochanter, gluteal tuberosity, and other regions with a protuberance structure as references. 

Typically, CT images are displayed on a monitor as a two-dimensional image that can be viewed as an image formed by three-dimensional spatial perspective projections. The figure’s information is subject to angular errors. In most cases, a three-dimensional coordinate system needs to be reconstructed. By locating the endpoint coordinates of pins in different sequences of CT images, Equation (1) can be used to calculate the displacement.

(1) $$d=\sqrt{{\left(x_{1}-x_{2}\right)}^{2}+{\left(y_{1}-y_{2}\right)}^{2}+{\left(z_{1}-z_{2}\right)}^{2}}\;\;$$

where (*x*_1_, *y*_1_, *z*_1_) and (*x*_2_, *y*_2_, *z*_2_) represent the coordinates of the same point in the two measurements, respectively.

### 2.3. Preprocessing of CT Images

**Figure 3:**
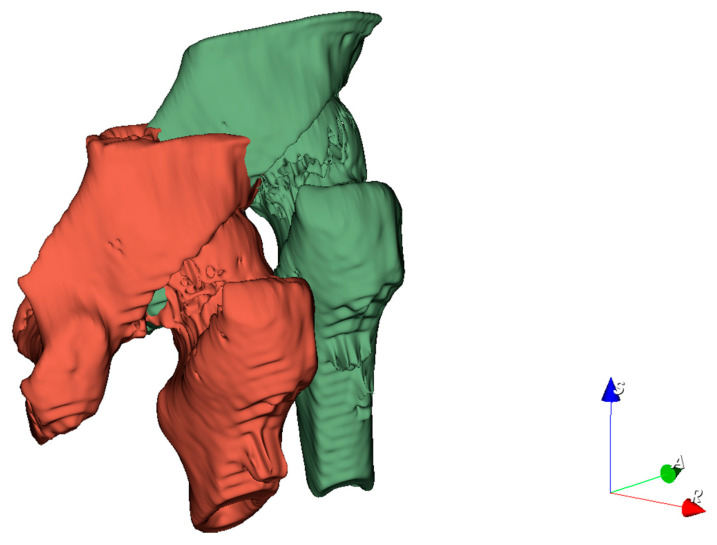
Models generated from different series of CT images are placed in the same coordinate system.

The generated 3D model based on the CT scan can be affected by the patient’s posture and position relative to the CT scanner during the CT recording. Besides, changes in skeletal health and initial orientation during the 3D reconstruction can also model the spatial position. These lead to the inability to directly calculate the position changes of the Hansson pins between the postoperation period and the one-year recovery period (Figure 3).

**Figure 4:**
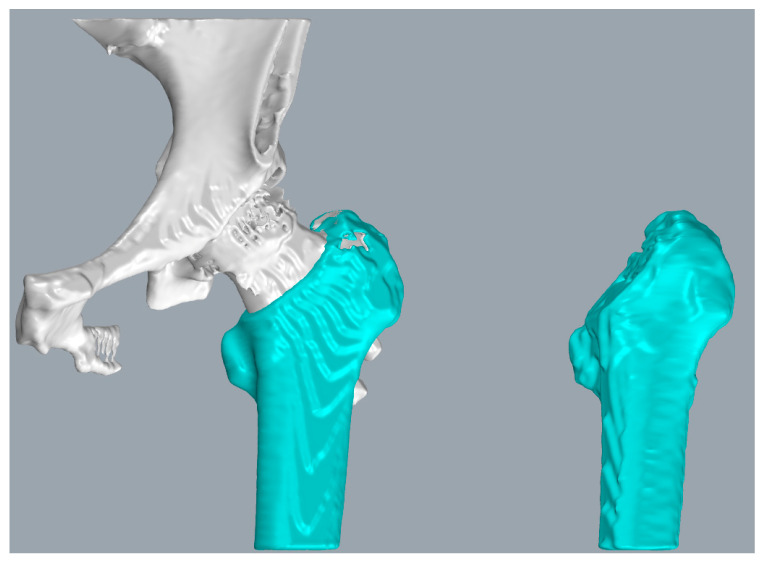
Selected partial femur model containing the greater trochanter and intertrochanteric crest, with the interior filled to use it as a reference.

To convert the three-dimensional models of pins postoperatively and one year after recovery into a uniform coordinate system for comparison, the reference should be selected as a relatively permanent structure, i.e., the reference does not change significantly during the one-year recovery process. Consequently, we chose the femur as the reference. According to the time sequence, the pins and femurs were reconstructed and transformed into 3D point clouds, respectively, based on the postoperative CT images and the CT images after the one-year recovery. In this paper, several model reconstruction techniques are employed to improve alignment accuracy and reduce the matching time. First, as shown in Figure 4, only the femur portion that did not contain the femoral head and the pins was used as a reference since the femoral neck fracture could cause a change of the femoral head position, and the pin position could also affect the matching accuracy. 

**Figure 5:**
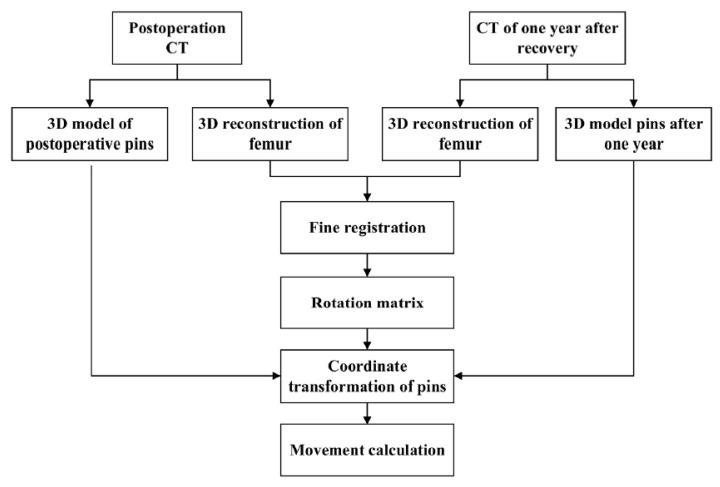
The process of 3D matching of the femur and the pins’ coordinate transformation.

Moreover, partial femur data also reduce the number of points in the model and reduce the calculation time during coordinate conversion. Besides, to reduce the point cloud noise during the alignment process, we filled the model’s interior during the model reconstruction process. Figure 5 presents the process of calculation.

Another technique that has been used is to roughly place the axis of the femoral part used for alignment in the z-direction of space during model reconstruction. In this way, the obtained model can be directly fine aligned. After applying the rotation matrix obtained from the alignment on the pins, the calculation of the implant shift can be performed.

### 2.4. Iterative Closest Point

**Figure 6:**
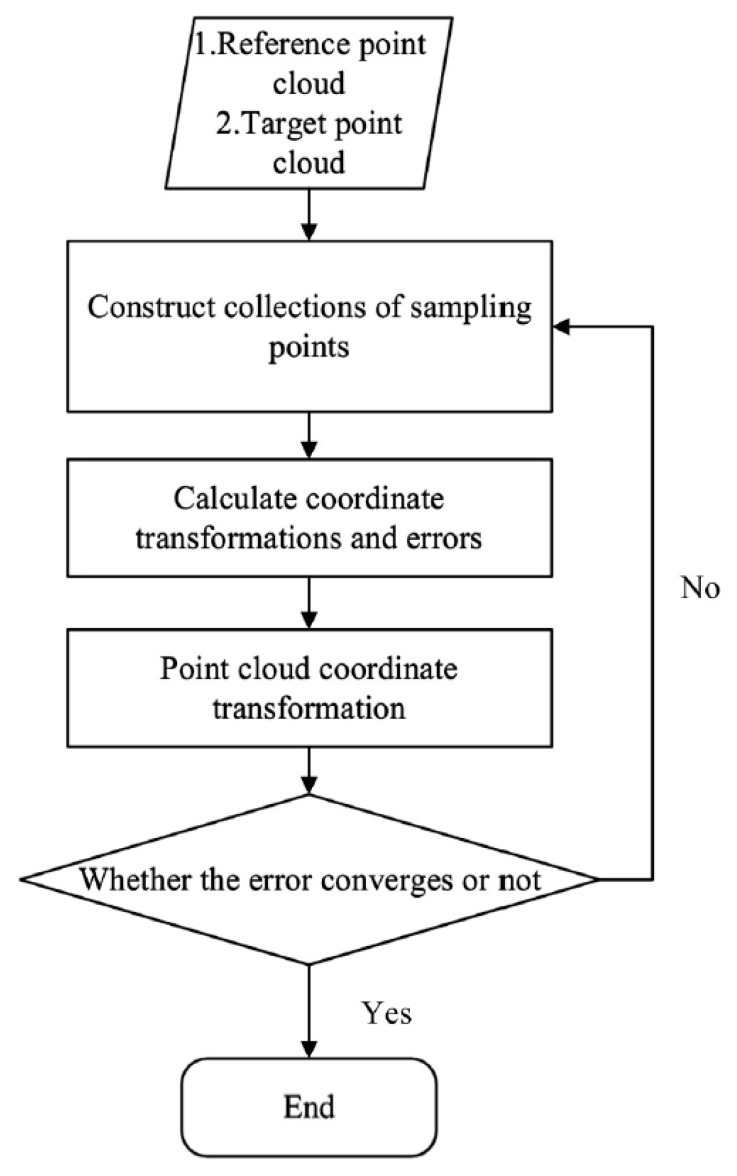
Process of performing alignment using the ICP algorithm.

The iterative closest point (ICP) algorithm is an iterative optimization method based on the least-square method that is used to solve the rigid transformation of the optimal registration of two sets of point clouds [19]. This algorithm aims to find the rotation and translation parameters between the point cloud to be matched and the reference point cloud using the specific geometric features. These parameters are used to transform the matched point cloud data. The iterations continue until the transformed results meet the requirements for convergence accuracy. The ICP algorithm execution process in this paper is shown in Figure 6.

The basic algorithm of the ICP is as follows: ***R****_n_* is defined as the rotation matrix after the Nth matching, and ***t*** is the translation vector.

Step 1: Take the two sets of point clouds ***P*** and ***Q*** as the initial point set of fine registration, where ***P*** is the point cloud to be matched and ***Q*** is the reference point cloud. ***P***’s center is flattened to coincide with the center of ***Q***, and the translation vector is ***t*_0_**.

Step 2: For each point ***p_i_*** in ***P***, we search for the corresponding point ***q_i_*** in ***Q***, from the corresponding points pairs, and then use the direction vector threshold to eliminate the wrong corresponding point pair. The product function is defined as

(2) $$E\left(R,t\right)=min\frac{1}{n}\overset{n}{\underset{i=1}{\sum }}∥q_{i}-R\cdot p_{i}{∥}^{2}$$

Then we compute the rotation matrix that minimizes the mean square of the distance.

Step 3: Point cloud ***P*** rotates according to the optimal solution in Equation (3), as follows.

(3) $$P_{n}=R_{n-1}\cdot P_{n-1},(n>=2)$$

The rotation matrix ***R*** can be solved by analyzing the covariance matrix between the corresponding point clouds.

Step 4: Determine whether the error *E*(***R***,***t***) is less than the preset value. If it is true, the iterations are stopped and failing, in which case Steps 2 and 3 are repeated until the iterations are completed. Then, the result can be expressed as

(4) $$R=\prod_{i=1}^{n}R_{i}$$

(5) $$t=R\cdot t_{0}$$

(6) $$P_{n}=R\cdot P+t_{0}$$

The ICP algorithm based on neighborhood features is widely used. Compared with the traditional algorithm, it can improve the point search rate and improve the matching points’ accuracy. The time cost of point cloud matching using the ICP algorithm depends on the femur part’s points number. It also depends on the computing power of the CPU. In this paper, the hardware information we used is shown in Table 1.

### 2.5. Experiment

#### 2.5.1. Data Preprocessing and Registration of Models

In this stage, we used a 3D Slicer [20] to reconstruct the 3D model. The 3D Slicer is a free open-source software for medical image analysis, visualization, and image-guided therapy research, which can be used on a variety of operating systems.

**Figure 7:**
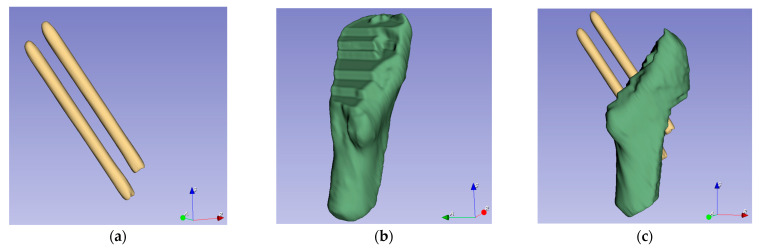

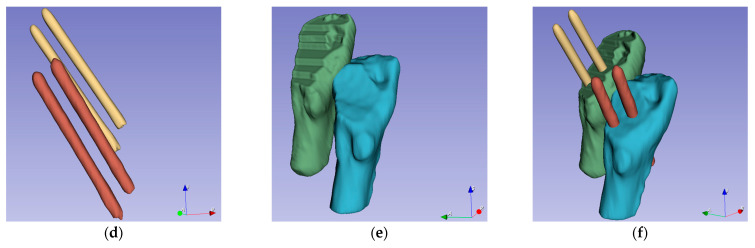
Using 3D Slicer to reconstruct 3D model of bone with the same initial direction: (**a**) The pins model reconstructed using CT images. (**b**) A part model of the femur. (**c**) Assembling the pins and femur from the same group of CT images in the same coordinate system. (**d**) Comparison of pins from different sets of dates. (**e**) The green model comes from postoperative data, and the blue model comes from CT images after a one-year recovery period. (**f**) All the models use the same initial direction.

We adopted the threshold tool and build-in Otsu algorithm to generate 3D models of the femur and the Hansson pin, respectively. Otsu is an algorithm to calculate the binarization segmentation threshold of an image proposed by Japanese scholar Otsu in 1979 [21]. Among the 3D models, the skeleton model’s minimum threshold range was 150, and the minimum threshold range of the pins were 1400. We applied these parameters and the 3D models were reconstructed. Figure 7 lists the models used in the next stage. Figure 7a,b presents how the pins and femur models were constructed, respectively, and that they share the same coordinate system. With the processing of 10 sets data, we obtained 10 sets of models. Then, fine registration was performed using the ICP method.

According to the different iterations’ registration results listed in Table 2, the registration accuracy remained stable when the iteration exceeded 125. The improvement in registration accuracy is less than 0.1% for an increase of 25 iterations. After the alignment was completed, we used the obtained rotation matrix and translation vectors to calculate the pin and femur positions.

#### 2.5.2. Measurement Based on 3D Coordinate System of CT Images

##### Manually Measure the Displacement Reference Value

**Figure 8:**
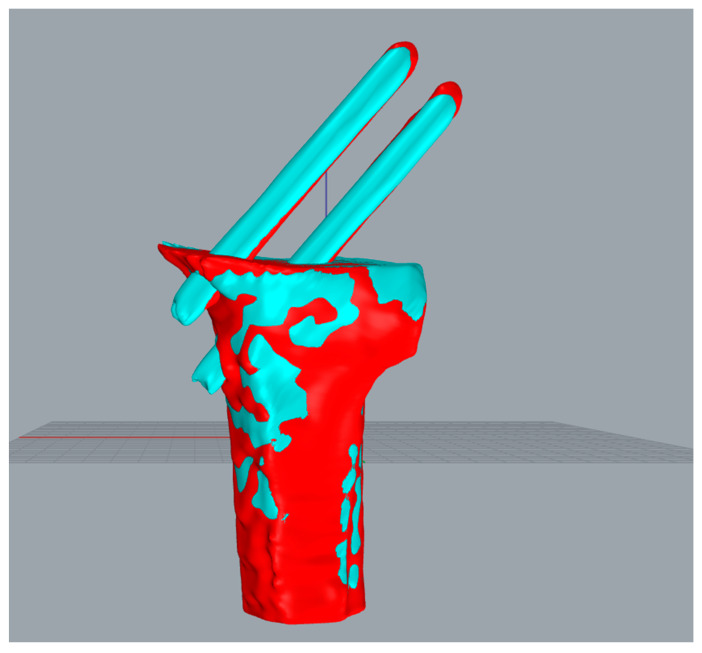
Manually matched and measured displacement of pins.

To verify the effectiveness and accuracy of the method proposed in this paper, we manually aligned the femur and pins models and measured the displacement of the pin endpoints. By repeating the operation three times, as we mentioned above, and calculating the average value, we obtained the reference values of actual pin migration used to verify the traditional method’s accuracy and the accuracy of the method proposed in this paper. Figure 8 explains that we imported two groups of 3D reconstruction models before and after a one-year recovery period into the Rhinoceros software [22], matched the two models artificially, and measured the displacement data of the corresponding point of the pins.

##### Measurement by the Traditional Method

**Figure 9:**
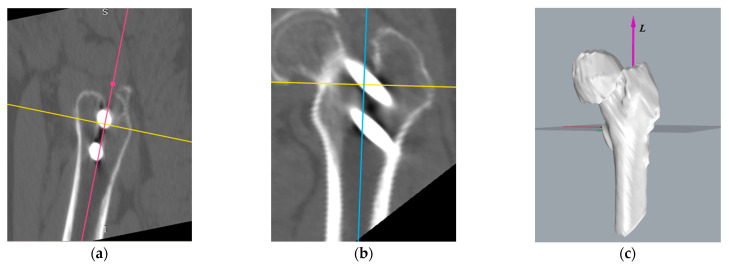
According to the projection of the medullary cavity centerline in plane 1 (red line) and plane 2 (blue line), we located the position of: (**a**) plane 1, (**b**) plane 2, and (**c**) the centerline in the 3D model.

The first step in creating a coordinate system is to locate the centerline of the medullary cavity, which hardly changes its relative position without significant damage to the corpus femoris. In the software RadiAnt [23], we performed multiplanar reconstruction to CT images to obtain three mutually perpendicular planes. By moving two of the planes, we found the medullary cavity’s projection, and located the centerline of the medullary cavity, line ***L***, according to the position information in two mutually perpendicular planes. The process of locating line ***L*** is shown in Figure 9. The midline of the medullary cavity was defined as the *z*-axis, with its positive direction pointing to the greater trochanter.

**Figure 10:**
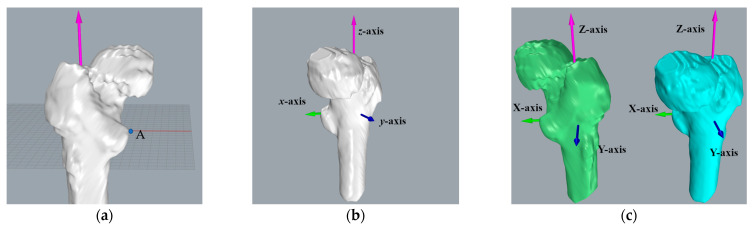
Establishing the right and left femoral coordinate system: (**a**) reference point A in the lesser trochanter region. (**b**) left femoral coordinate system, and (**c**) comparison of the left and right femoral coordinate systems.

In Figure 10a shows the process of creating the *x*-axis in the left femur. We located the farthest point A from the *z*-axis in the lesser trochanter region. The line that goes through point A and vertical to the *z*-axis is the *x*-axis, with the positive direction pointing to point A. Additionally, the intersection point O of the *x*-axis and *z*-axis is the coordinate origin. We defined the line passing through the origin and perpendicular to the *x*-axis and *z*-axis as the *y*-axis. The positive direction of the *y*-axis is the direction of the cross product of the *z*-axis and the *x*-axis. Similarly, the coordinate system in the right femoral model, as shown in Figure 10c, was established.

**Figure 11:**
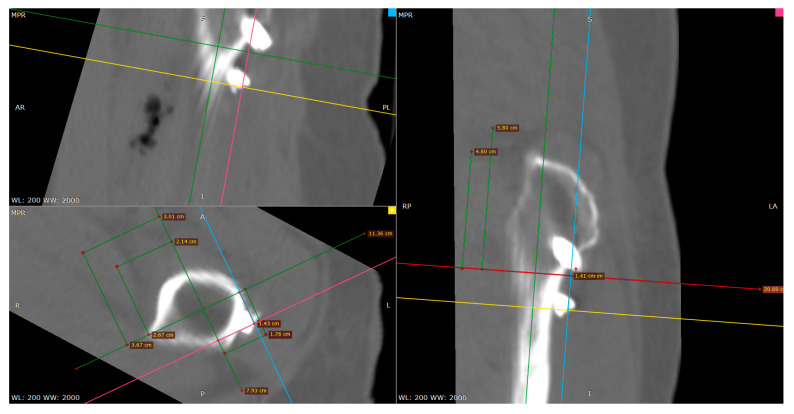
Using software RadiAnt to measure the position of the Hansson pin.

Figure 11 presents the established coordinate system, the process of measuring the proximal pin’s coordinates, and the distal pin’s endpoints.

#### 2.5.3. Calculation of Pin Displacement Based on Point Cloud Matching

**Figure 12:**
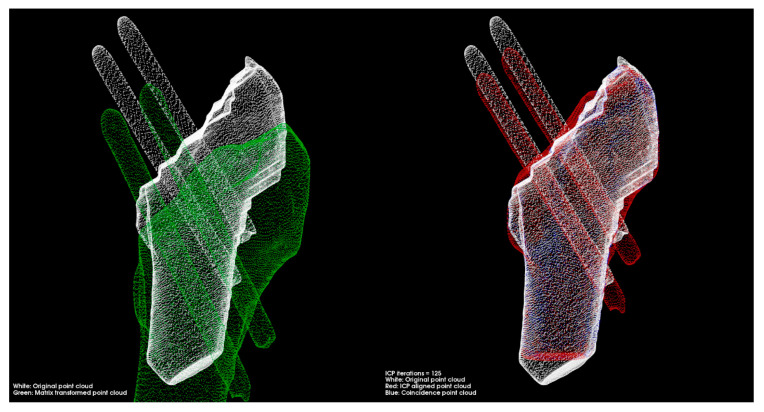
Performing position transformation on the pins and femur after registration with 125 iterations.

Figure 12 exposes the position of the pins and femur after registration with 125 iterations. The white point cloud is the data measured after the surgery, and the green one is the point cloud to be matched based on the data after a one-year recovery. Then the green point cloud transforms into the red part using position transformation.

**Figure 13:**
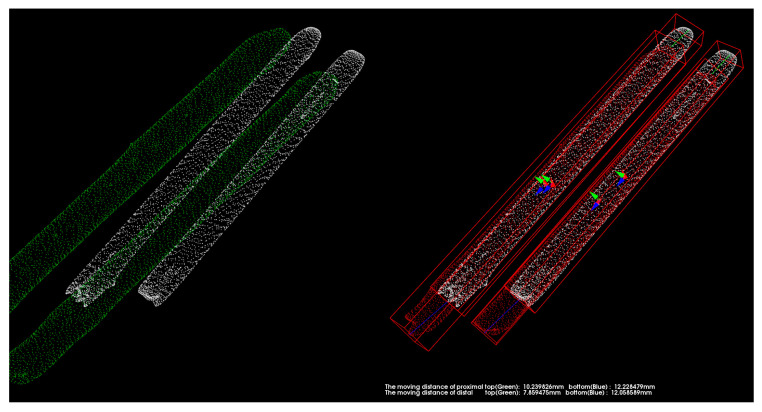
Visualization of pins’ movement and calculation of moving distance and rotation angle.

We eliminated the femur part and quantitatively calculated the pins’ movement in the three-dimensional space. As shown in Figure 13, the principal component analysis was used to calculate the direction vector for obtaining relative angles, drawing the bounding box for the pins, and obtaining the endpoint coordinates. Meanwhile, the actual length of pins listed in Table 3 was used to calibrate the length scale of the point cloud data.

After femur registration, each femur model’s alignment effect was evaluated on the basis of the distance from each point in the converted femur point cloud to the nearest point in the benchmark point cloud. This subsection used the maximum value, the average value, the proportion of points whose distance was less than 2 mm, and the proportion of points whose distance was less than 0.5 mm as evaluation criteria. 

**Figure 14:**
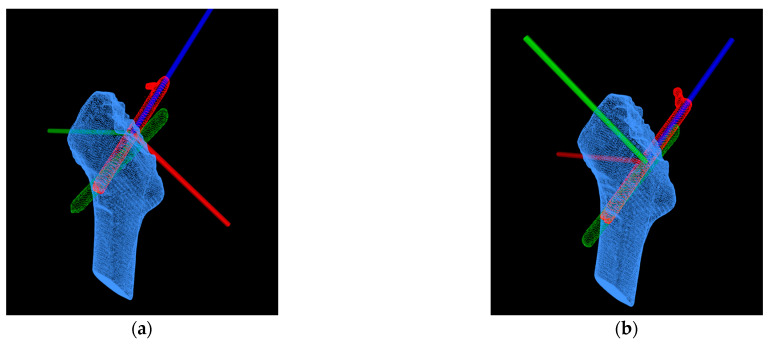
Converting the aligned point cloud data to the new coordinate system: (**a**) the coordinate system of the proximal pin; (**b**) the coordinate system of the distal pin.

Additionally, for a more precise analysis of the Hansson pin’s movement in the femur, we transformed the aligned model into a new coordinate system. Figure 14 describes the new coordinate system of the proximal pin and the distal pin after transformation. The red pin depicts the point cloud of the Hansson pin postoperatively, and the green pin depicts the point cloud of the Hansson pin one year after surgery.

According to the 3D point cloud of the postoperative proximal pin in Figure 14a, we fit its central axis, the blue axis, as the *z*-axis of the new coordinate system, pointing to the top of the Hansson pin as the positive direction. The center of the point cloud of the pin served as the origin of the coordinate system. Passing the origin, the *y*-axis was established parallel to the hook pin, and the positive direction was defined as the direction of the pin elongation. Furthermore, the *y*-axis vector and *z*-axis vector’s cross product was used as the vector of the red *x*-axis. Moreover, the direction was determined by the result of the cross product. Similarly, a new coordinate system for the distal pin in Figure 14b was built.

## 3. Results

### 3.1. Result of Registration

Table 2 describes the result of registration with different iterations. From the proportion of points whose distance to the corresponding point was less than 0.5 mm, when the iterations reached 125, the optimal result could be obtained.

Table 4 shows the time used when the iterations were set to 125 for the 3 cases in the table that were randomly selected from the 10 cases. The time increased by five milliseconds for each additional point.

**Figure 15:**
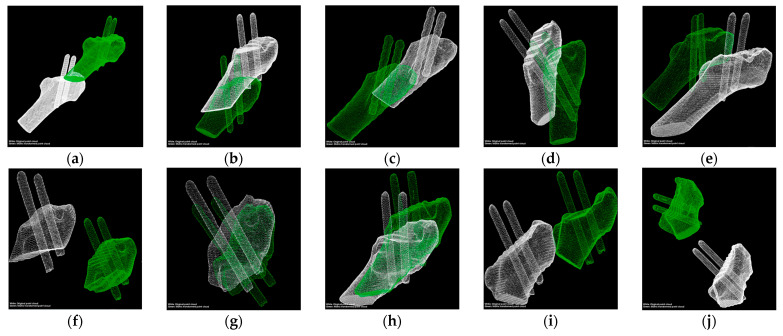
3D point clouds preprocessing. (**a**–**j**) are the point clouds of the 10 cases without performing alignment, respectively. The white point cloud is generated based on postoperative CT images, and the green point cloud is generated based on CT images scanned after the one-year recovery period.

Figure 15 presents the data preprocessing results, and the 10 groups of models based on the CT images. The models’ main directions in each group were the same and could be used directly for fine registration.

**Figure 16:**
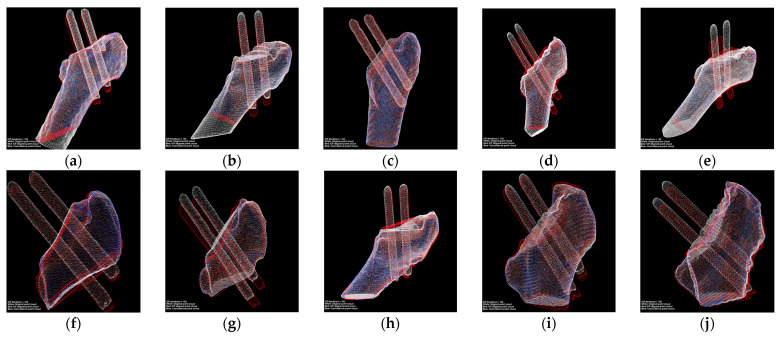
Transformation using the matrix obtained from the registration. (**a**–**j**) are the point clouds after fine registration for each of the 10 cases. The white point cloud is generated based on the postoperative CT images. The red point cloud is transformed form the point cloud generated based on the CT images scanned after one year. The blue point clouds are the coincident points after point cloud registration.

Figure 16 displays the results after the position transformation using the matrix obtained from the registration. The red model can be regarded that the green model in Figure 15 transformed their coordinate systems to the coordinate systems of white models.

**Figure 17:**
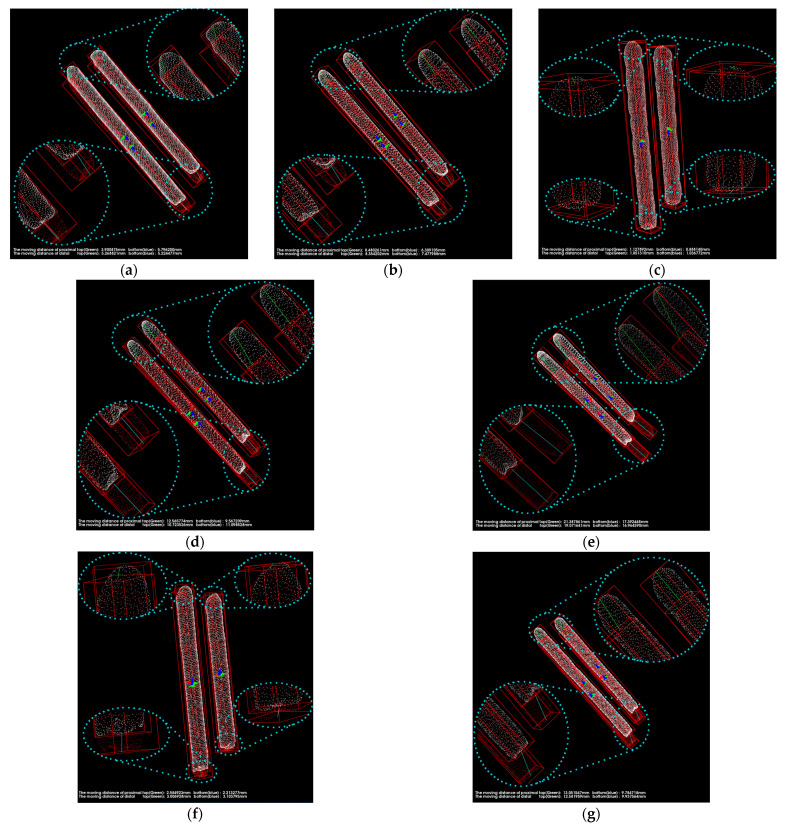

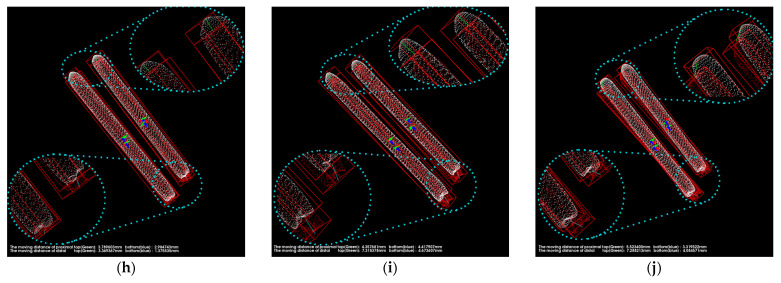
The results of the calculation of the pins’ migration. (**a**–**j**) are the point clouds of pins transformed to the same coordinate system for each of the 10 cases, respectively. The white point cloud is generated based on the postoperative CT images. The red point cloud is transformed form the point cloud generated based on the CT images scanned after one year.

As shown in Figure 17, the pins and the bounding boxes of each group were transformed into the same coordination system. The green lines in the image represent the distance traveled by the top vertexes of the pins, and the blue lines describe the moving distance of the lower vertexes of the pins. What is more, the movement of each pin can be perceived from various perspectives. The data we obtained are listed in Table 5, and all data are retained with two significant digits.

Table 6 shows the displacement of the Hansson pin in each direction in the new coordinate system that is discussed in Section 2.5.3.

### 3.2. Results of Evaluation

In this paper, we defined the corresponding points in the post-alignment point cloud and the reference point cloud with distances less than 0.5 mm as coincident points, indicated by blue dots in Figure 16. The average and maximum distances of the corresponding points in all experimental cases are listed in Table 7. Besides, the proportion of corresponding points at a distance of less than 2 mm and the percentage of overlapping points were used to evaluate the degree of overlap between the two point clouds.

Table 8 shows that in the first case, the coordinates of the Hansson pins’ endpoints and the pins’ length calculated using these coordinates were measured for the same patient at two different times using the conventional method. The results show that the length of pins computed using this method had an error between 0.6 mm and 1 mm, which proves that the error of the method proposed in this paper is limited.

Table 9 compares the results obtained using the conventional method, the method proposed in this paper, and the manual measurement in the first case. Compared with the traditional method, our method has a clear advantage and improvement in the measurement results’ accuracy.

**Figure 18:**
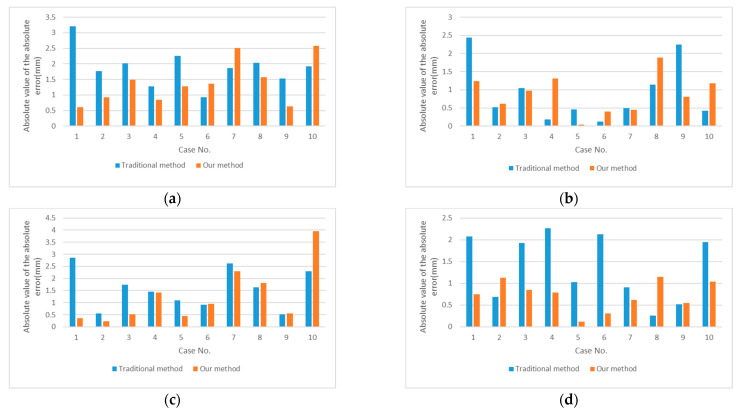
Comparison of the absolute value of the absolute error of the results obtained using the traditional method and the method proposed in this paper: (**a**) Comparison of top endpoint displacement results on the proximal pin. (**b**) Comparison of bottom endpoint displacement results on the proximal pin. (**c**) Comparison of top endpoint displacement results on the distal pin. (**d**) Comparison of bottom endpoint displacement results on the distal pin.

Figure 18 illustrates the comparison of the results’ absolute error values using the conventional method and our method. Where Figure 18a,b shows the displacement of the top and bottom endpoints of the proximal pin, and Figure 18c,d shows a comparison of the distal pin measurements.

## 4. Discussion

The incidence of proximal femoral fractures has increased significantly with the population’s aging, which has occurred far more among women than men [17]. The preferred treatment for stable femoral neck fractures is internal fixation, where pins or screws are the main components use for internal fixation [24]. Implant stability is critical to the success of internal fixation procedures and femoral healing. Traditional methods of evaluating implant stability mainly use biological simulation and finite element analysis, which have limitations and cannot comprehensively evaluate the stability of implants. CT medical images can provide displacement data of the implant inside the patient’s body and can most directly evaluate the stability of the implant under real force conditions. However, the current clinical practice mainly relies on the manual measurement of displacement data by surgeons, which imposes a significant workload on surgeons, and the accuracy of the measurement results is highly dependent on the experience of surveyors. In this research, we propose a method to measure implants’ movement after internal fixation for patients suffering proximal femur fractures, and this method is based on 3D point cloud registration.

In Reference [14], Schopper, Clemens, et al. evaluated two internal fixation systems by simulating the pelvis’s pressure on the femur during human gait and measured the force and displacement of the implants in the frontal and sagittal planes. In another study, the authors used finite element models to implement biomechanical simulations of five different configurations of internal fixation implants [15]. In both studies, only the pelvis’s pressure on the femur was simulated during the standing phase of the patient’s gait, ignoring the interaction forces of the muscles and other organs. More critically, they did not study the internal fixation implants in the fixation effect throughout the patient’s postoperative recovery. In daily life, patients spend most of their time sleeping and sitting, which can have a cumulative effect on the implant position and its stability. The method proposed in this paper directly utilizes authentic postoperative CT medical images of the patient as the basis for the study. The results in Table 6 show the displacement of the pins after the patient has experienced one year postoperatively. This method addresses the limitations of traditional biomechanical simulation methods in simulating the complex environment.

We found that the ICP algorithm can provide high precision in fine alignments. However, with the number of points increasing in the point cloud, the time consumed per iteration also grows. To solve a problem that requires a long time, we downsized the point cloud or removed parts of the 3D model with less feature quantity. In our experiment, we used a part of the femur for registration, and satisfactory results were obtained. According to the outcomes listed in Table 4, when the number of iterations was set to 125, the time used for registration increased by five milliseconds for each additional point. Therefore, we can reduce the registration time by reducing the number of model points while still meeting the registration accuracy requirements. In this study, we also found that a portion of the corresponding points in the aligned model had a distance greater than 2 mm, and this fraction ranged from 3% to 19%. The possible reason for is that, in the process of 3D reconstruction, some non-femoral regions are identified as the femur as the threshold value is the same as the femur, resulting in some irregular points existing in the generated femur. Since these points do not belong to the femur, the corresponding points cannot be found, but the nearest points are matched, which affects the experimental results. The extraction of boundary information from point clouds and the development of point cloud filtering methods are expected to improve the experimental results.

The results in Table 9 demonstrate that, in the current study, the newly proposed method showed a significant improvement in the error of the measurement results compared to the method in which the surgeon establishes a coordinate system in the CT images for measurement. The conventional measurement method relies strongly on the expert’s experience in establishing the coordinate system and selecting the target location, which is susceptible to subjective factors.

Another important finding based on the results listed in Table 6 was that the Hansson pin moved gradually downward in the femur, whose coordinate system was built and is described in Section 2.5.3. In 10 cases, all pins moved along the Hansson pin’s central axis, toward the lateral aspect of the femur. For seven of the cases, the distal pins were displaced to varying degrees in the opposite direction of the hook pin extension, and the displacements of the top ends were greater than the displacements of the bottom ends, indicating that the pins also rotated to the opposite direction of the hook pin elongation during the displacement process. 

In Figure 7e, the smoothness of the two femoral models’ outer surface without the femoral head is not consistent; this is because the two 3D femoral models originated from different CT image sequences. These two sequences were scanned one year apart and used different scanning equipment and scanning parameters. The green model was derived from CT medical images after an internal fixation surgery with an image layer spacing of 3 mm. The blue model was derived from CT images after a one-year recovery period with an image layer spacing of 1.5 mm. While cutting the 3D model, the images with a large layer spacing produced significant faults at the incision, which did not affect the reference registration.

The major limitation of this study is that we used the built-in threshold tool of a 3D Slicer to implement the automatic 3D reconstruction of CT medical images. The reconstructed 3D model contained the whole femur and the connected part of the pelvis, which needed to be artificially segmented in order to obtain the alignment object, as shown in Figure 4. In addition, it was necessary to manually remove the interfering points generated during the 3D reconstruction process to avoid the influence of these interfering points on the registration results. The average time required to manually remove the interference points and segment the alignment object was 120 s. The time required to measure the implant displacement depended on the number of sampling points of the alignment object and can be controlled to be less than 200 s. The total time required to complete an implant displacement was approximately 270 s. Since the preprocessing of the 3D point cloud data in this study was independent of the displacement calculation stage, the automatic segmentation of the reference part could also be achieved by other methods in future studies to reduce the time consumption. In addition, the purpose of this study was to verify the effectiveness of the proposed method, therefore there was no optimization in our study for the registration time. The main time of the displacement calculation stage was spent on the alignment of the reference 3D point cloud, and we could reduce the time consumption by decreasing the number of sampling points.

## 5. Conclusions

In this investigation, the aim was to present a method based on point cloud matching for evaluating the stability of internal fixation implants in femur fractures during patient recovery. This method was based on the Hansson pins analysis and is widely applicable to the analysis of other implants used for fixation. We reconstructed the femur and implant using CT images of patients at different times after internal fixation surgery. A portion of the femur with no femoral head was selected as a reference, and the pin-point clouds from different periods were converted to the same coordinate system to calculate the endpoint displacement of the corresponding pin. Meanwhile, a new coordinate system based on the pin axis and extension direction of the hook pin was used to evaluate the Hansson pin as a feature.

Since the same initial orientation was chosen for the same set of data in the 3D reconstruction model, the femur’s rough alignment was avoided, which reduced the alignment time and improved the accuracy.

Furthermore, the measurement error in our study was limited, and the method yielded satisfactory results.

## Figures and Tables

**Table 1:** Hardware information.

Hardware	Configuration
CPU	Core i7-2700k 3.50GHz
Memory	16GB
Operating system	Windows10

**Table 2:** Result of accuracy comparison with different iterations.

Iterations	Points Whose Distance Is Less Than 0.5 mm		
	Case 1	Case 2	Case 3
25	22.83%	5.77%	31.63%
50	42.22%	10.87%	57.82%
75	37.75%	21.31%	57.27%
100	37.50%	50.17%	57.18%
125	37.50%	49.12%	57.19%
150	37.50%	49.08%	57.19%
175	37.50%	49.09%	57.20%
200	37.50%	49.08%	57.20%
225	37.51%	49.08%	57.22%
250	37.51%	49.08%	57.24%

**Table 3:** The length of pins and information of patients in each case.

Case No.	Length of Pins		Gender	Age
	Proximal	Distal		
1	80	90	female	79
2	85	95	female	76
3	80	95	female	81
4	90	100	female	65
5	80	90	female	78
6	75	90	female	85
7	85	100	female	79
8	85	90	female	77
9	80	90	female	73
10	80	90	female	67

**Table 4:** Time consumed for different size point cloud registrations.

Case No	Number of Points in the Model	The Time Spent (min)
1	47,354	3.80
2	17,878	1.69
3	26,357	1.75

**Table 5:** Result of relative angles and movement of the pins.

Case No.	Proximal Pin			Distal Pin		
	Relative Angle (°)	Top Movement (mm)	Bottom Movement (mm)	Relative Angle (°)	Top Movement (mm)	Bottom Movement (mm)
1	0.93	3.90	5.80	1.69	5.27	5.22
2	1.19	8.48	6.31	0.94	8.35	7.48
3	1.08	1.13	0.89	1.38	1.85	1.04
4	1.85	12.57	9.57	1.96	10.72	11.10
5	11.26	21.39	17.39	3.19	19.07	16.96
6	3.02	2.58	2.21	2.51	3.01	3.10
7	9.00	13.05	9.78	7.58	12.58	9.94
8	2.79	3.79	2.98	2.16	3.37	1.38
9	2.61	4.36	4.42	4.39	7.32	4.67
10	5.17	5.52	3.32	5.45	7.29	4.08

**Table 6:** Displacement of the Hansson pins.

Case No.	Endpoint	Movement of Proximal Pin (mm)			Movement of Distal Pin (mm)		
		*x*-Axis	*y*-Axis	*z*-Axis	*x*-Axis	*y*-Axis	*z*-Axis
1	top	2.07	0.20	−3.39	0.03	−2.68	−4.61
	bottom	0.73	0.33	−5.85	−0.40	−0.02	−5.27
2	top	0.02	0.57	−8.67	0.58	−1.07	−8.47
	bottom	1.02	−0.91	−6.32	−0.39	0.19	−7.64
3	top	−0.02	−1.10	−0.31	1.67	−0.03	−0.85
	bottom	−0.56	0.33	−0.62	−0.61	0.40	−0.75
4	top	−2.66	−0.98	−12.63	−2.15	−0.68	−10.89
	bottom	0.03	0.23	−9.85	1.37	−1.18	−11.37
5	top	8.55	−6.27	−19.36	−1.35	−3.43	−18.95
	bottom	−4.08	3.57	−17.12	0.69	1.15	−17.12
6	top	1.18	2.27	−0.59	−2.44	0.27	−1.84
	bottom	−1.45	−0.8	−1.52	1.46	−0.8	−2.69
7	top	−6.15	−6.38	−9.76	7.15	−4.76	−9.26
	bottom	1.11	4.9	−8.51	−5.43	−0.61	−8.35
8	top	1.23	1.76	−3.18	−1.96	1.37	−2.46
	bottom	−1.86	−1.02	−2.15	0.33	−1.16	−0.71
9	top	−0.93	−1.66	−4.03	5.06	−1.89	−5.16
	bottom	−0.44	2.04	−4.01	−1.72	−0.12	−4.45
10	top	−0.71	4.73	−2.96	−5.82	−0.11	−4.45
	bottom	−0.63	−2.59	−2.08	2.72	−0.12	−3.07

**Table 7:** Evaluation results.

Case No.	Iteration (s)	The Average Distance (mm)	The Max Distance (mm)	Points Whose Distance Is Less Than 0.5 mm	Points Whose Distance Is Less Than 2 mm
1	125	0.91	11.87	44.30%	93.77%
2	125	0.92	11.54	49.12%	90.33%
3	125	0.57	4.16	57.19%	97.44%
4	125	1.15	7.79	28.71%	85.33%
5	125	1.02	7.68	37.45%	87.64%
6	125	0.86	4.14	33.45%	94.19%
7	125	1.44	8.01	20.15%	81.17%
8	125	1.02	7.18	38.33%	88.36%
9	125	1.10	5.87	31.82%	85.71%
10	125	0.96	5.67	33.39%	90.80%

**Table 8:** Results measured by traditional methods.

Pin		Coordinates After the Operation				Coordinates After One-Year Recovery			
		*x*	*y*	*z*	Length (mm)	*x*	*y*	*z*	Length (mm)
Proximal	top point	21.4	−26.7	58	80.04	21.1	−19.5	57.5	81.34
	endpoint	−16.2	17.6	2.96		−18.3	19.5	−2.02	
Distal	top point	30.1	−36.7	47.7	90.03	30.1	−30.3	46.5	91.98
	end point	−14.3	12.1	−14		−15.9	16.3	−18.1	

**Table 9:** Comparison results.

Pin	Endpoint	Manual Measurement (mm)	Traditional Method		Our Method	
			Displacement (mm)	Relative Error	Displacement (mm)	Relative Error
Proximal	top point	3.30	7.22	118.79%	3.90	18.21%
	bottom point	4.56	5.73	25.66%	5.80	27.11%
Distal	top point	4.92	6.51	32.32%	5.27	7.09%
	bottom point	4.48	6.08	35.71%	5.22	16.62%

## Data Availability

The data on the patients included in this clinical study are not publicly available due to ethical and privacy reasons.
